# Identifying and confirming quantitative trait loci associated with heat tolerance at flowering stage in different rice populations

**DOI:** 10.1186/s12863-015-0199-7

**Published:** 2015-04-22

**Authors:** Changrong Ye, Fatima A Tenorio, May A Argayoso, Marcelino A Laza, Hee-Jong Koh, Edilberto D Redoña, Krishna SV Jagadish, Glenn B Gregorio

**Affiliations:** International Rice Research Institute, DAPO Box 7777, Metro Manila, 1301 Philippines; Seoul National University, Seoul, 151-921 South Korea; Present address: Mississippi State University, P. O. Box 197, Stoneville, MS 38776 USA

**Keywords:** Global warming, Heat stress, Quantitative trait locus, Spikelet fertility, Rice

## Abstract

**Background:**

Climate change is affecting rice production in many countries. Developing new rice varieties with heat tolerance is an essential way to sustain rice production in future global warming. We have previously reported four quantitative trait loci (QTLs) responsible for rice spikelet fertility under high temperature at flowering stage from an IR64/N22 population. To further explore additional QTL from other varieties, two bi-parental F_2_ populations and one three-way F_2_ population derived from heat tolerant variety Giza178 were used for indentifying and confirming QTLs for heat tolerance at flowering stage.

**Results:**

Four QTLs (*qHTSF1.2*, *qHTSF2.1*, *qHTSF3.1* and *qHTSF4.1*) were identified in the IR64/Giza178 population, and two other QTLs (*qHTSF6.1* and *qHTSF11.2*) were identified in the Milyang23/Giza178 population. To confirm the identified QTLs, another three-way-cross population derived from IR64//Milyang23/Giza178 was genotyped using 6K SNP chips. Five QTLs were identified in the three-way-cross population, and three of those QTLs (*qHTSF1.2*, *qHTSF4.1* and *qHTSF6.1*) were overlapped with the QTLs identified in the bi-parental populations. The tolerance alleles of these QTLs were from the tolerant parent Giza178 except for *qHTSF3.1*. The QTL on chromosome 4 (*qHTSF4.1*) is the same QTL previously identified in the IR64/N22 population.

**Conclusion:**

The results from different populations suggest that heat tolerance in rice at flowering stage is controlled by several QTLs with small effects and stronger heat tolerance could be attained through pyramiding validated heat tolerance QTLs. QTL *qHTSF4.1* was consistently detected across different genetic backgrounds and could be an important source for enhancing heat tolerance in rice at flowering stage. Polymorphic SNP markers in these QTL regions can be used for future fine mapping and developing SNP chips for marker-assisted breeding.

**Electronic supplementary material:**

The online version of this article (doi:10.1186/s12863-015-0199-7) contains supplementary material, which is available to authorized users.

## Background

Global warming caused by greenhouse gases has a huge effect on sustaining agricultural development. Since the early 20th century, the earth's mean surface temperature has increased by 0.8°C, with about 0.6°C of this hike occurring since 1980 [1]. Global mean surface temperatures for 2081–2100, relative to 1986–2005, is likely to increase by 0.3°C to 1.7°C for the lowest and by 2.6°C to 4.8°C for the highest greenhouse gas emission scenarios [2]. This temperature change will not be regionally uniform [3]. It is virtually certain that, in most places, there will be more hot and few cold temperature extremes as global mean temperature increases. Of the 13 warmest years since 1880, 11 occurred from 2001 to 2011 (i.e. every year starting 2001). Of the more recent records, 2005 was the second warmest year behind 1998, with 2003 and 2010 tied for the third warmest year, while 2011 was the warmest La Niña year in the period from 1950 to 2011 [4,5]. These drastic changes in temperature in recent years have caused more frequent occurrence of extreme-weather events such as heat waves and drought, with serious consequences on rice yield.

Rice yield losses due to high temperature have been reported in many tropical and subtropical countries, such as in Pakistan, India, Bangladesh, China, Thailand, Japan, Australia and the U.S. [6-9]. Significant yield losses have also been predicted by using different crop models. Short term predictions indicated that, by 2030, rice production in South Asia could decrease by up to 10% [10]. Medium to long term predictions, i.e. by 2080, estimated rice yields in developing countries to decrease by 10% to 25%, on average, while yields in India could drop by 30% to 40% [11]. By 2100, rice and maize yields in the tropics are expected to decrease by 20–40% because of higher temperatures without accounting for the decrease in yields as a result of drought enhanced by temperature increases [12]. Spatial model simulation indicated that yield of *boro* rice in Bangladesh could decrease by 20% and 50% by 2050 and 2070, respectively [13], and on average, rice yields could be reduced by up to 33% by 2081–2100 [14]. As the global temperature continuously rises, incorporation of heat tolerance in new rice varieties is therefore, becoming more and more important in rice breeding programs.

Flowering is the most sensitive stage to high temperature in the rice life cycle [15,16]. High temperature of over 35°C at flowering stage increases pollen and spikelet sterility, which leads to significant yield losses, low grain quality, and low harvest index [6,7,17-20]. Large cultivar variation exists in the spikelet sensitivity to high temperature damage, and the primary cause of this cultivar variation in high temperature (heat) tolerance at flowering is the number of viable pollen grains shed on the stigma, resulting from the changes in the extent of anther dehiscence [21], which directly affect the spikelet fertility and grain yield [17]. Thus, spikelet fertility under high temperature has been widely used as a screening index for heat tolerance at reproductive stage [19].

In recent years, rice varieties tolerant to high temperature have been identified [17,22,23]. QTL mapping studies for heat tolerance at flowering stage have been conducted on various rice populations [24-31]. However, while QTLs for heat tolerance at flowering stage have been mapped on almost all rice chromosomes, improving heat tolerance in rice varieties using the identified genetic resources and QTLs has not yet been achieved. Further studies on QTL mapping using diverse genetic resources and populations are needed for addressing the means to enhance heat tolerance in rice. We have developed a precise method for phenotyping rice heat tolerance and employed the Illumina GoldenGate SNP assay technology for mapping QTLs for heat tolerance using IR64/N22 population [30]. The phenotyping protocol was shown to be repeatable and reliable in our ongoing fine mapping of the previously identified QTLs (*qHTSF1.1* and *qHTSF4.1*) using different backcross populations. Here, we report the identification of QTLs for rice heat tolerance at the flowering stage using three other populations derived from Giza178, identified to be one of the most heat-tolerant rice varieties at flowering. The main objectives of this study were to identify QTLs controlling heat tolerance in Giza178 at flowering stage using different susceptible recipients, and to confirm the identified QTLs in an advanced population.

## Methods

### Developing mapping populations

To select novel heat tolerant donors for QTL mapping, we evaluated the heat tolerance of 455 rice varieties from different countries [23,32] and a set of over 40 promising varieties for heat tolerance at booting and flowering stages [23]. Preliminary analysis showed that the spikelet fertility of IR64, a heat sensitive variety, decreased significantly even with 1°C increase between 36–39°C. At 39°C, the spikelet fertility of IR64 dropped to less than 5%, but the tolerant varieties, such as N22 and Giza178, maintained fertility levels of over 60% (unpublished data). IR64 (susceptible) and Milyang23 (moderately susceptible) were crossed with Giza178 (tolerant) to develop two different mapping populations. IR64 is a mega-variety cultivated widely in Asia with low to moderate tolerance to high temperature at flowering stages [33,34]. Milyang23 is a Korean variety developed from the cross IR1317-316-5-1/IR24, while Giza178 is an Egyptian variety developed from the cross Giza175/Milyang49. Both Milyang23 and Giza178 have *Indica* and *Japonica* introgressions in their earlier pedigrees. The F_1_ plants were self-pollinated to produce F_2_ populations for QTL identification. No hybrid sterility was observed in the F_1_ plants. Due to the limited space in the growth chambers, relatively small populations were used to identify QTL with larger effects, rather than using large population to detect small-effect QTL. One hundred F_2_ progenies from each cross were phenotyped for heat tolerance at flowering stage in 2012; and 86 and 96 progenies from IR64/Giza178 and Milyang23/Giza178 populations, respectively, were genotyped using a custom 384-plex Illumina GoldenGate SNP assay (*Indica-Indica*).

Based on the phenotyping results of the Milyang23/Giza178 F_2_ population, the top ten heat tolerant plants were crossed with IR64 to develop a three-way breeding population (Additional file 1). The three-way F_1_ lines (IR64//Milyang23/Giza178) were preliminarily screened in a temperature-controlled phytotron (35/24°C day/night temperature, 80% relative humidity) at flowering stage. From each line, 2–3 plants with good agronomic traits and high spikelet fertility were harvested, and 10 seeds from each plant were grown in a net house for phenotyping in 2013 as described below.

### Phenotyping of F_2_ progeny

The seeds of the parents IR64, Milyang23, Giza178 and the F_2_ progenies were germinated and sown in plastic pots (L × W × H = 9.5 × 9.5 × 8.5 cm with draining holes) filled with natural clay loam soil, one plant per pot. The pots were randomly arranged in trays (L × W × H = 75 × 50 × 20 cm) to ensure that plants were grown under the same water and nutrient conditions. The plants were grown in a net house under natural temperature and sunlight as described by [30]. Twenty-one days after sowing, a young leaf (about 10 cm long) from each plant was collected and frozen for DNA extraction, and 10 g of PerfectGro® composite fertilizer 14-14-14 (AFC Fert. & Chem., Inc) was applied to each tray. The position of the pot in the tray was changed within the tray every week to reduce any potential micro-climate effects of light and temperature, and to avoid interweaving of the roots that protruded from the bottom of the pots. Only the main stem and two tillers were maintained, all the other tillers were removed to prevent overcrowding. When each plant started heading, the plant was transferred into a new tray and moved into an indoor growth chamber (IGC, Thermoline, Australia). The temperature regime in the IGC was the same as described by [30] with 6 hours of high temperature (38°C) each day during the flowering time (08:30–14:30). After 14 days of temperature exposure the plants were moved back to the net house and grown to maturity. The date of high temperature treatment commencement (heading date) was recorded. At physiological maturity, the number of fully filled spikelets (including partially filled) and empty spikelets were counted. The mean spikelet fertility of the three panicles maintained in each pot was used to evaluate the heat tolerance of the plant.

### Genotyping using SNP markers

Genomic DNA of IR64, Milyang23, Giza178 and all the F_2_ plants was extracted using SDS extraction buffer (100 mM Tris, 50 mM EDTA, 500 mM NaCl, 1.25% SDS and 1% v/v 2-mercaptoethanol) and chloroform/isoamylalcohol (24:1) solution followed by ethanol precipitation. The RNA was digested by RNase at 37°C for 30 minutes. The final concentration of the DNA samples was normalized to 50 ng/μl for genotyping. The SNP genotyping was done by using Illumina BeadXpress 384-plex SNP plates GS0011861 (customized for *Indica-Indica*) for the bi-parental populations and Illumina Infinium 6 K SNP beadchip for the -three-way F_2_ population. The custom oligo pool assay (OPA) containing 384 well-distributed SNPs per assay was designed by Cornell University [35,36] from a high quality subset of the SNPs discovered in 20 diverse *O. sativa* landraces [37]. The 6 K beadchip was designed for SNP fingerprinting, high-resolution mapping and genome-wide SNP scans by Dr Susan MacCouch’s group at Cornell University. It contains about 6,000 bead types, and 5,274 SNPs are high quality in the dataset, 247 of which are overlapped with the BeadXpress 384-plex SNP set (*Indica-Indica*). The PCR amplification and hybridization were carried out following the GoldenGate genotyping assay for VeraCode manual protocol (Illumina, San Diego, CA). If the SNP genotype is the same as in IR64, it was coded as AA, otherwise it was coded as BB, and the heterozygote was coded as AB.

### Statistical analysis

The average spikelet fertility of different genotypes was compared by one-way ANOVA using MINITAB V14.0 (Minitab Inc.). The VeraScan raw data from BeadXpress Reader was initially analyzed using the GenomeStudio software V1.1.0 (Illumina Co.) and Alchemy [38]. The generated report and map file were used for QTL analysis by using composite interval mapping (CIM) in QGene V4.3.8 [39]. The genetic distance between SNP markers was estimated from the physical map based on the genomic sequence available at GRAMENE (www.gramene.org), with genetic distance (cM) = Physical distance (kb)/260. CIM was performed using the standard model with a walk speed of 2 cM. Cofactor selection was set at auto. Permutation tests [40] were performed for each trait with composite interval mapping and 1,000 permutations. For the 6 K SNP genotyping, we firstly checked the marker-trait association by using the Trait Analysis by Association Evolution and Linkage (TASSEL) program [41]. The sites were filtered at a maximum count of 150 of the 167 F_2_ plants, which accounts for sites where 90% of the lines have a call and a minimum frequency of 0.05 for the minor allele. The above criteria resulted in 4497 filtered sites. Among them, 1373 SNPs are polymorphic among the parents. These polymorphic SNPs were used for association analysis using general linear model (GLM) [42]. In this study, we set a significant threshold of p < 0.0001 (−log_10_*p*-value > 4.0). The genotypic and phenotypic data were then used for QTL mapping using Qgene software as described above. The identified QTLs were named using the CGSNL nomenclature [43]. The correlation between QTL alleles and spikelet fertility, and the interaction among QTLs were further analyzed by using MINITAB V14.0 (Minitab Inc.).

## Results

### Heat tolerance of the populations

Differences in spikelet fertility of IR64 (22.3 ± 11.1%), Milyang23 (44.5 ± 11.8%) and Giza178 (62.2 ± 12.7%) were significant following high temperature treatment in 2012 (Table 1). The spikelet fertility of the F_2_ progeny of IR64/Giza178 population ranged from 0 to 82.1% (Mean = 29.6, SD = 24.0), while that of Milyang23/Giza178 population ranged from 0.2 to 88.7% (Mean = 38.6, SD = 25.7). The distributions of both F_2_ populations were slightly skewed to the lower spikelet fertility side i.e. with more susceptible progeny.

**Table 1:** **Spikelet fertility (%) of the parental varieties and F**_**2**_ **populations**

**Year**	**Parent/Population**	**Plants**	**Mean**	**StD**	**Range**	**Skewness**	**Kurtosis**
	IR64	9	22.3	11.1	5.7 - 34.6	−0.59	−1.12
	Milyang23	9	44.5	11.8	26.1 - 57.9	−0.31	−1.42
2012	Giza178	17	62.2	12.7	40.6 - 78.8	−0.19	−1.39
	IR64/Giza178 F_2_	86	29.6	24	0.0 - 82.1	0.42	−1.19
	Milyang23/Giza178 F_2_	96	38.6	25.7	0.2 - 88.7	0.17	−1.18
	IR64	15	21.4	11.5	8.0 - 38.5	0.54	−1.34
2013	Milyang23	15	49.4	8.3	36.2 - 59.6	−0.53	−1.39
	G178	15	63.6	6.9	50.8 - 72.6	−0.35	−0.85
	IR64//MY23/G178 F_2_	166	59.2	20.9	0.0 - 90.6	−0.74	−0.26

In 2013, similar spikelet fertility was observed for the parental varieties IR64 (21.4 ± 11.5%), Milyang23 (49.4 ± 8.3%) and Giza178 (63.6 ± 6.9%) after high temperature treatment. This confirmed the heat stress response of IR64 (susceptible), Milyang23 (moderately susceptible) and Giza178 (tolerant). The spikelet fertility of the three-way F_2_ population ranged from 0 to 90.6% (Mean = 59.2, SD = 20.9). The average spikelet fertility of the population was higher than those of the two F_2_ populations in 2012.

### QTLs identified from IR64/Giza178 and Milyang23/Giza178 populations

**Figure 1:**
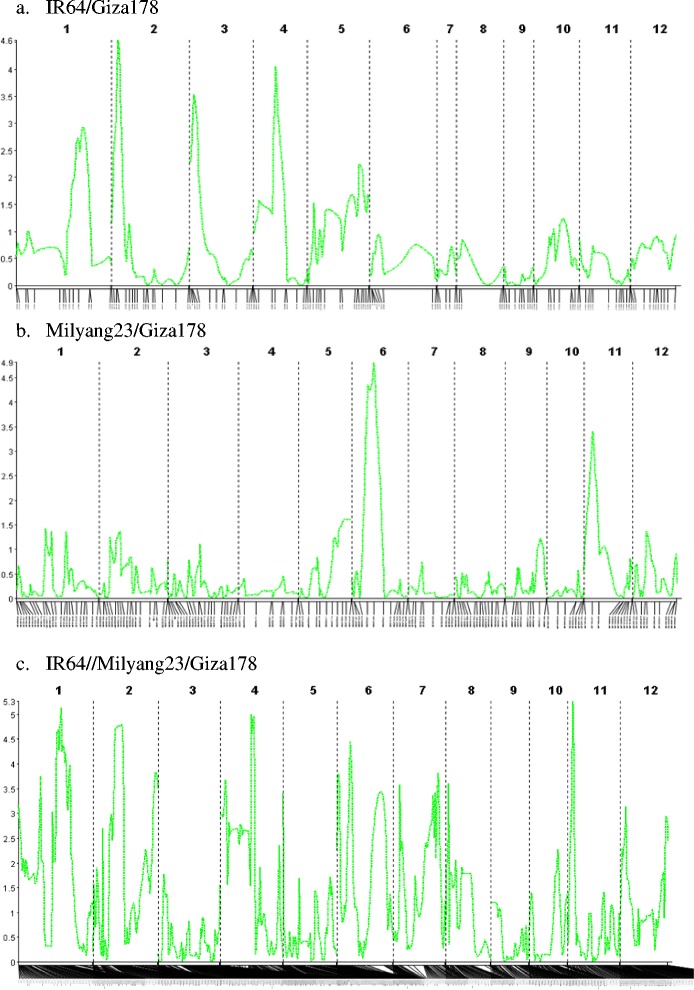
QTLs for spikelet fertility under high-temperature conditions from different populations. QTL analysis was accomplished through composite interval mapping (CIM) using QGene V4.3.8. Permutation α_0.05_ is 3.64, 3.52 and 3.86 for population **a)** IR64/Giza178, **b)** Milyang23/Giza178 and **c)** IR64//Milyang23/Giza178, respectively.

**Figure 2:**
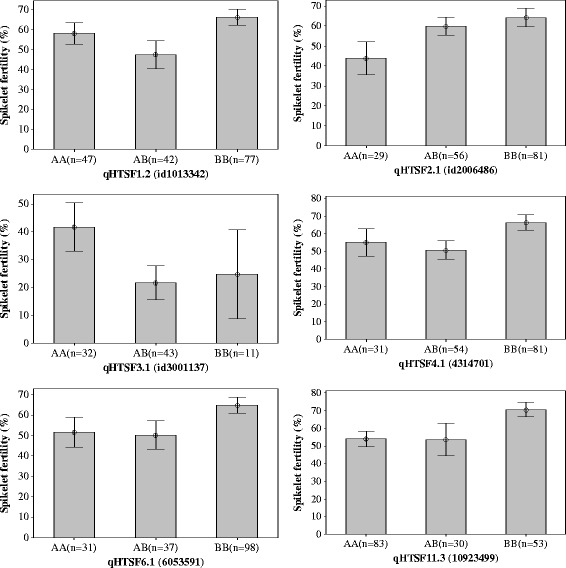
Interval plot of spikelet sterility of F_2_ progeny using SNP markers close to QTLs (peak of LOD score) of *qHTSF1.2*, *qHTSF2.1*, *qHTSF3.1*, *qHTSF4.1*, *qHTSF6.1* and *qHTSF11.3*. The interval bar shows 95% confidence interval of the mean. *qHTSF3.1* is from the IR64/Giza178 population, whereas the others are from the three-way population IR64//Milyang23/Giza178. Genotype AA = IR64, AB = heterozygote, BB = Milyang23 or Giza178.

Among the screened 384 SNP markers, 133 (34.6%) markers showed polymorphism between IR64 and Giza178. The results from CIM showed four potential QTLs (*qHTSF1.2*, *qHTSF2.1*, *qHTSF3.1* and *qHTSF4.1*) on chromosome 1, 2, 3 and 4 (Figure 1a). These QTLs explained around 15 - 22% of the variation in spikelet fertility caused by high temperature treatment, with both additive and dominance effects. The tolerant alleles for the QTLs are from the tolerant parent Giza178, except for *qHTSF3.1* (Table 2). At marker id3001137 on chromosome 3, the mean spikelet fertility of AA genotype (41.8 ± 24.4%) was significantly higher than that of BB genotype (24.8 ± 23.9%) and heterozygote AB (21.7 ± 20.0%), and the spikelet fertility of BB and AB genotypes are not significantly different. The allele increasing spikelet fertility comes from the susceptible parent IR64 controlled by a recessive gene (Figure 2).

**Table 2:** **QTLs identified for spikelet fertility under high temperature**

**Population**	**Locus name**	**Chr.**	**Position (interval) (cM)**	**LOD**	**Additive effect**	**Dominance effect**	**R2 (%)**	**Tolerant allele**
	*qHTSF1.2*	1	120.5 (103.5-129.5)*	2.94	16.8	−18.0	14.6	Giza178
I/G	*qHTSF2.1*	2	13.8 (4.8-19.8)	4.58	5.2	−29.3	21.8	Giza178
	*qHTSF3.1*	3	9.5 (1.5-17.5)	3.54	−9.7	−14.2	17.3	IR64
	*qHTSF4.1*	4	68.5 (63.5-80.5)*	4.06	16.5	−14.0	19.5	Giza178
M/G	*qHTSF6.1*	6	46.5 (27.5-59.5)*	4.81	17.2	−0.3	20.6	Giza178
	*qHTSF11.2*	11	33.3 (25.3-42.3)	3.43	14.2	23.0	15.2	Giza178
	*qHTSF1.2*	1	89.0 (80.0-109.0)*	5.16	4.1	−14.6	13.3	Giza178
	*qHTSF2.2*	2	61.0 (43.0-63.0)	4.80	10.4	5.9	12.5	Giza178
I//M/G	*qHTSF4.1*	4	66.0 (66.0-73.0)*	5.00	11.7	−16.0	13.0	MY23 or Giza178
	*qHTSF6.1*	6	29.5 (24.5-31.5)*	4.45	6.7	−9.8	11.6	Giza178
	*qHTSF11.3*	11	14.6 (11.6-14.6)	5.38	8.3	−8.7	13.9	Giza178

*QTL intervals were overlapped.

^#^Population I/G = IR64/Giza178, M/G = Milyang23/Giza178, I//M/G = IR64//Milyang23/Giza178. Permutation α_0.05_ is 3.64, 3.52 and 3.86 for population I/G, M/G and I//M/G, respectively.

Among the same 384 SNP markers, 178 (46.4%) markers showed polymorphism between Milyang23 and Giza178. The results from CIM showed two potential QTLs (*qHTSF6.1* and *qHTSF11.2*) on chromosome 6 and 11 (Figure 1b). The QTL on chromosome 6 (LOD = 4.81) was located between SNP markers id6004481 and id6008973 at about 12.09 Mb. This QTL explained 20.6% of the variation in spikelet fertility caused by high temperature treatment. The QTL on chromosome 11 (LOD = 3.42) was located between SNP markers id11002639 and id11003924 at about 8.66 Mb, which explained 15.2% of the variation in spikelet fertility caused by high temperature treatment. The tolerant alleles for *qHTSF6.1* and *qHTSF11.2* are from the tolerant parent Giza178 (Table 2).

### QTL identified from IR64//Milyang23/Giza178 population

For the genome-wide SNP scans using the 6 K beadchip, a dataset with 5,274 SNPs was obtained. Among them, 4,492 high quality SNPs have a call on 90% of the progeny and 1373 SNPs are polymorphic among the parental varieties. Most of the SNPs are evenly distributed on the chromosomes, with only a few small gaps on chromosome 6, 7 and 8. The largest gap is about 8.95 Mb on chromosome 6.

Through Cladogram analysis using TASSEL, one plant showed unusual segregation with others. This plant was removed, and a total of 166 plants were included in subsequent analysis. Results from the GLM analysis showed that some SNPs on chromosome 1, 2, 4, 6 and 11 are significantly associated with spikelet fertility under high temperature (−ln^(p-value)^ > 4.0) (Additional file 2).

To conduct CIM analysis using Qgene, only the genotypes of the 1373 polymorphic SNPs were used. The results showed that five QTLs are potentially controlling the spikelet fertility under high temperature conditions (Figure 1c), which is consistent with the results from association analysis by using TASSEL. The LOD score of these QTLs ranged from 4.45 to 5.38 (permutation threshold α_0.05_ = 3.86, α_0.01_ = 4.60). These QTLs explained 11.6 – 13.9% of the variation in spikelet fertility caused by high temperature stress treatment (Table 2).

The spikelet fertility of different genotypic classes (AA, AB and BB) were significantly different for the SNP markers close to the peaks of the QTLs. *qHTSF2.1* is controlled by a dominant gene (d = 5.9), the other QTLs are all controlled by recessive genes (Figure 2). The tolerant alleles of all QTLs are from the tolerant parent Giza178.

**Figure 3:**
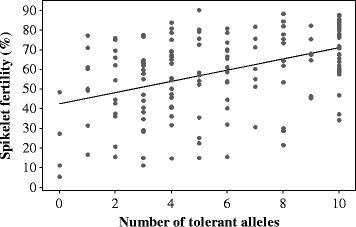
Accumulative effect of QTLs identified in the IR64//Milyang23/Giza178 population. Number of alleles was coded as AA = 0, AB = 1 and BB = 2. A score of 0 means no allele from Giza178 in all the five QTL regions, whereas a score of 10 means the plants have alleles from Giza178 in all 5 heat-tolerance QTL regions. Correlation r = o.441, p < 0.0005, n = 166.

There is an accumulative effect of the five QTLs identified in IR64//Milyang23/Giza178 population. The more QTLs introduced, the higher tolerance the plant has (Figure 3). The plants with all the 5 QTLs are generally more tolerant to high temperature, their mean spikelet fertility (72.2 ± 13.2%) is similar or even slightly higher than the tolerant parent Giza178 (63.6 ± 6.9%).

**Figure 4:**
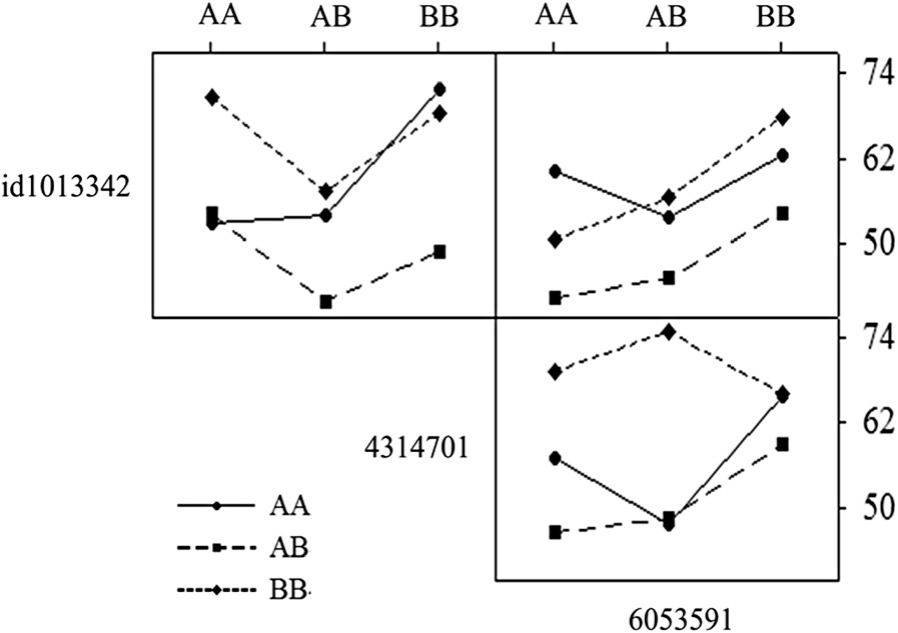
Interaction among *qHTSF1.2*, *qHTSF4.1* and *qHTSF6.1* in IR64//Milyang23/Giza178 population. AA is IR64 genotype, AB is heterozygote and BB is Milyang23 or Giza178 genotype. id1013342, 4314701 and 6053591 are the SNP markers close to the peak of the LOD score. The numbers at the right side are spikelet fertility in percent (%).

Among the five QTLs identified, *qHTSF1.2* and *qHTSF6.1* are overlapped with the QTLs identified in IR64/Giza178 and Milyang23/Giza178 populations, and *qHTSF4.1* is located within the QTL region identified in IR64/Giza178 population. The other two QTLs (*qHTSF2.2* and *qHTSF11.3*) are located nearby but not overlapped with the QTLs identified on the same chromosomes from the bi-parental populations (Table 2). There are interactions among the confirmed three QTLs, *qHTSF1.2* can maintain relatively high spikelet fertility without *qHTSF4.1*, and *qHTSF4.1* can maintain relatively high spikelet fertility without *qHTSF6.1*. The spikelet fertility even slightly decreased with both QTLs. The interaction of *qHTSF1.2* and *qHTSF6.1* could enhance heat tolerance when both QTLs are working together (Figure 4).

## Discussions

Giza178 is an *Indica-Japonica* rice variety from Egypt, which showed consistent heat tolerance in our experiments in the past few years (Tenorio et al. 2013). By using the F_2_ population derived from the IR64/Giza178 and Milyang23/Giza178 crosses, six QTL were identified that are associated with spikelet fertility under high temperature conditions. However, different QTLs were identified in the two populations, though most of the tolerant alleles are from Giza178 except that for QTL on chromosome 3. QTLs for heat tolerance were located on chromosome 1, 2, 3 and 4 in IR64/Giza178 population, while those in Milyang23/Giza178 population were located on chromosome 6 and 11. The LOD scores of *qHTSF1.2*, *qHTSF3.1*, and *qHTSF11.2* are slightly lower than the threshold from permutation, but these QTLs explained 14.6-17.3% of the variation of spikelet fertility under heat stress, and *qHTSF1.2* was confirmed to be overlapped with a QTL identified in the three-way population. Thus, they were considered as potential QTLs. Among these six QTLs, three were confirmed in the three-way-cross population. Different QTLs were identified in different populations suggests that a strong background (or population) dependence exists among the mapping populations, and some of the important tolerant alleles may not be distinguished in a bi-parental population. To identify most of the tolerant alleles in a donor variety, it is necessary to cross the donor with different susceptible recipients. Nested association mapping (NAM) populations, in which a set of diverse lines is crossed with a common reference line, can be used as next generation mapping populations [44]. Alternatively, a multi-parental population, such as multi-parent advanced generation inter-cross (MAGIC) population [42,45], can be used for identifying tolerant alleles associated with the trait by association mapping, which provides a platform for the discovery and characterization of genes responsible for complex traits like heat tolerance.

The tolerant alleles for the identified QTLs are from Giza178 except *qHTSF3.1* on chromosome 3. For QTL *qHTSF3.1*, the spikelet fertility of the genotypes of the SNP marker close to the QTL showed that the heat tolerant allele is from the susceptible parent IR64. A QTL (*qhr3-1*) for heat tolerance was previously identified on the short arm of chromosome 3 [24]. QTL *qhr3-1* may be located in the same interval as *qHTSF3.1*. However, *qHTSF3.1* was not identified in the three-way cross population, this QTL may not be contributing to increasing heat tolerance when some of the heat tolerance alleles from Giza178 were introduced into IR64 background.

The QTLs on chromosome 1 (*qHTSF1.2*) and 11 (*qHTSF11.2* and *qHTSF11.3*) are different from those previously identified (*qHTSF1.1* and *qHTSF11.1*) in the IR64/N22 population [30]. But *qHTSF1.2* and *qHTSF11.2* are located in similar regions with *qhr1* and *qhr11-1* reported by [24]. The QTLs *qHTSF2.1*, *qHTSF6.1* and *qHTSF11.3* are newly identified loci related to spikelet fertility under high temperature stress conditions.

In the QTL region of *qHTSF4.1* on chromosome 4, there is no polymorphism between Milyang23 and Giza178. Both Milyang23 and Giza178 have the tolerant allele for this QTL. This also explained why *qHTSF4.1* was not identified in the Milyang23/Giza178 population. The same 384-plex SNP markers were used for genotyping IR64/N22 [30] and IR64/Giza178 populations. The QTLs identified in both populations are located in the same chromosomal regions and both of them are closely linked to SNP marker id4005120. We named this QTL as *qHTSF4.1*, the same name with the QTL identified from IR64/N22 population [30]. QTL *qHTSF4.1* explained a similar percentage of the variation in spikelet fertility under high temperature conditions, and showed very similar genetic effects in both IR64/Giza178 and IR64/N22 populations. This QTL is also confirmed in the three-way-cross population IR64//Milyang23/Giza178. At this locus, the mean spikelet fertility of Giza178 genotype (AA = 66.5 ± 19.5%) was significantly higher than those of IR64 genotype (aa = 55.1 ± 21.4%) and heterozygote (Aa = 50.8 ± 19.1%), and there was no significant difference between IR64 genotype and heterozygote (Figure 2). The allele increasing spikelet fertility is from the tolerant parent Giza178 and *qHTSF4.1* is controlled by a recessive gene. The same QTL controlling spikelet fertility under high temperature was also identified in a similar chromosomal region [29]. This confirms that *qHTSF4.1* exists in different heat tolerant rice varieties; and therefore, could be considered important for increasing rice spikelet fertility under high temperature. Our fine mapping results also showed that *qHTSF4.1* increased spikelet fertility under high temperature conditions in different backcross populations. In a BC_5_F_2_ population with clean background of IR64, QTL *qHTSF4.1* increased spikelet fertility by about 15% (Additional file 3).

Three QTLs identified on chromosome 1, 4 and 6 in the bi-parental populations were confirmed in the three-way population. These QTLs explained 11.6-13.0% of the variation in spikelet fertility caused by high temperature treatment. The additive effect of each QTL ranged from 4.1 to 11.7, indicating that heat tolerance at flowering stage is controlled by several QTLs with small genetic effects. There is a positive correlation between spikelet fertility and the number of alleles from Giza178 in the 5 QTL regions (r = 0.441, p < 0.0005, n = 166). The effects of these QTLs are accumulative (Figure 3). Increasing the number of QTLs in the plant will therefore, increase its heat tolerance. Thus, it is possible to pyramid these QTLs in breeding programs to improve the heat tolerance of new breeding lines.

By using two small populations (IR64/Giza178 and Milyang23/Giza178) with low marker density, we were able to identify most of the QTLs with relatively higher genetic effects. However, the QTL intervals are quite large (3.9-8.3 Mb), and the genetic effects of these QTLs were overestimated. When high-density SNP markers were used for genotyping a larger three-way population IR64//Miyang23/Giza178, big gaps were overcome. However, the recombination in the QTL regions of *qHTSF1.2* and *qHTSF2.1* was still low, hindering the possibility of getting higher resolution for these QTLs. On the other hand, the confidence intervals of the QTLs on chromosome 4 (*qHTSF4.1*), 6 (*qHTSF6.1*) and 11 (*qHTSF11.3*) were reduced to around 0.8-1.8 Mb. Though there are still many genes in the QTL regions, interestingly some heat shock protein (HSP) related genes are located in the QTL regions of *qHTSF1.2* (*Os01g42190*) and *qHTSF6.1* (*Os06g13060* and *Os06g14490*). HSPs play fundamental roles in protecting plants against abiotic stresses, such as heat stress [46]. This may be important information for us to identify candidate genes for heat tolerance in the future.

QTLs for rice heat tolerance at flowering stage have been mapped on all chromosomes by using various rice populations and high temperature treatment methods [24-31] (Additional file 4). However, as the confidence intervals for some of the QTLs are quite large, it is still difficult to use linked markers of those QTLs for marker-assisted selection in breeding programs. At the same time, the additive effect of each QTL is low. Introducing one or a few QTLs into a plant may not be sufficient to significantly increase its heat tolerance. Introgression of more tolerant alleles will be needed to achieve higher heat tolerance. Furthermore, after high temperature treatment, the overall spikelet fertility of the Milyang23/Giza178 population (38.6 ± 25.7%) was higher than that of IR64/Giza178 population (29.6 ± 24.0%). This is because Milyang23 is more tolerant to high temperature than IR64. This suggests that using heat tolerant varieties as parents in a breeding program will increase the frequency of heat tolerant progeny in the populations. The average spikelet fertility of the three-way population was higher than those of the two bi-parental populations. This is because the three-way F_1_ population was preliminarily selected after exposure to temperature closer to known critical thresholds of 35°C in the phytotron. It suggests that selection for heat tolerance at early generations is effective. Since it is difficult to accommodate large number of breeding lines in temperature-controlled conditions, it is possible to do the pre-selection using F_2_ or backcrossed F_1_ populations as an effective and practical strategy to save space. In this study, we identified large number of SNP markers in the QTL regions. It is possible to design specific SNP chips using those SNP markers, such as Fluidigm Dynamic Array, and using SNP chips for marker assisted selection. This will help to improve the efficiency of heat tolerance breeding.

## Conclusions

In this study, we identified 8 QTLs controlling spikelet fertility under high temperature, and three of them were indentified in at least two populations. The results from different populations suggest that heat tolerance in rice at flowering stage is controlled by several QTLs with small effects and stronger heat tolerance could be attained through pyramiding validated heat tolerance QTLs. QTL *qHTSF4.1* was consistently detected across different genetic backgrounds and could be an important source for enhancing heat tolerance in rice at flowering stage. Polymorphic SNP markers in these QTL regions can be used for future fine mapping and developing SNP chips for marker-assisted breeding. We are developing backcross populations to introduce the QTLs into IR64 background for further validation and fine mapping.

## Additional files

Additional file 1:
**Identifying and confirming QTL for rice heat tolerance Additional file 1.** Population development for identification and validation of QTLs for rice heat tolerance. Additional file 2:
**Identifying and confirming QTL for rice heat tolerance Additional file 2.** Manhattan plot for spikelet fertility under high-temperature conditions using 1,373 SNP markers. Additional file 3:
**Identifying and confirming QTL for rice heat tolerance Additional file 3.** Spikelet fertility of different genotypes in BC2F2, BC3F3 and BC5F2 populations of IR64/N22 cross. All plants were treated at 38 for 14 days during flowering. AA is IR64 genotype (without qHTSF4.1), AB is heterozygote, BB is N22 genotype (with qHTSF4.1). Additional file 4:
**Identifying and confirming QTL for rice heat tolerance Additional file 4.** List of published heat tolerance QTLs.

## Abbreviations

CIM: Composite Interval Mapping
cM: Centimorgan
LOD: Logarithm of odds ratio
QTL: Quantitative trait locus
QTLs: quantitative trait loci
SNP: Single Nucleotide Polymorphism
