# Supplementary figures and images for: Identifying and confirming quantitative trait loci associated with heat tolerance at flowering stage in different rice populations
Source: BMC Genet. 2015 Apr 22;16:41. doi: 10.1186/s12863-015-0199-7 (PMC4415243; doi:10.1186/s12863-015-0199-7)

## Slide 1
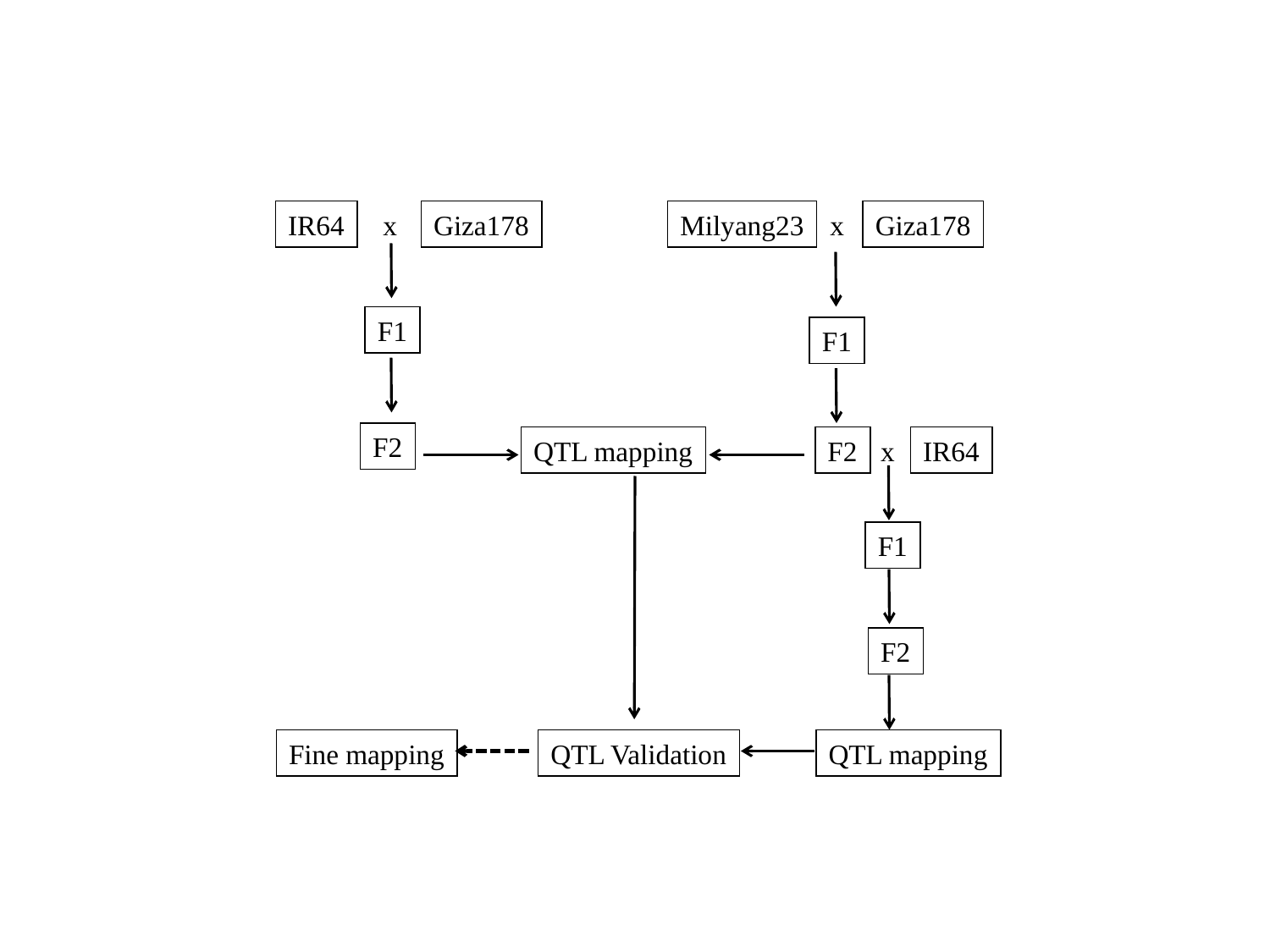

IR64
x
Giza178
Milyang23
x
Giza178
F1
F1
F2
QTL mapping
F2
x
IR64
F1
F2
Fine mapping
QTL Validation
QTL mapping

Supplement: Additional file 1: — Identifying and confirming QTL for rice heat tolerance Additional file 1. Population development for identification and validation of QTLs for rice heat tolerance. [file 12863_2015_199_MOESM1_ESM.pptx]

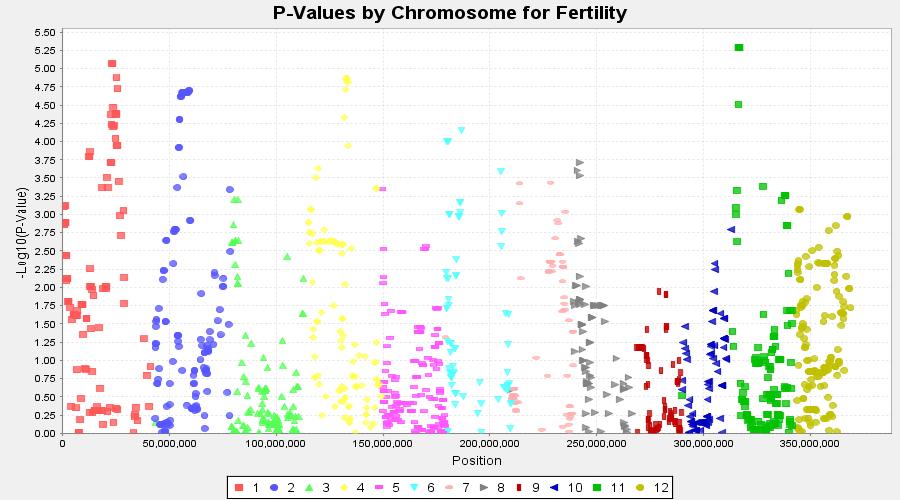

Supplement: Additional file 2: — Identifying and confirming QTL for rice heat tolerance Additional file 2. Manhattan plot for spikelet fertility under high-temperature conditions using 1,373 SNP markers. [file 12863_2015_199_MOESM2_ESM.jpg]
