# Supplementary material for: Identifying and confirming quantitative trait loci associated with heat tolerance at flowering stage in different rice populations
Source: BMC Genet. 2015 Apr 22;16:41. doi: 10.1186/s12863-015-0199-7 (PMC4415243; doi:10.1186/s12863-015-0199-7)
Supplement: Additional file 3: — Identifying and confirming QTL for rice heat tolerance Additional file 3. Spikelet fertility of different genotypes in BC2F2, BC3F3 and BC5F2 populations of IR64/N22 cross. All plants were treated at 38 for 14 days during flowering. AA is IR64 genotype (without qHTSF4.1), AB is heterozygote, BB is N22 genotype (with qHTSF4.1). [file 12863_2015_199_MOESM3_ESM.docx]

BC2F2

BC3F3

BC5F2
